# Analysis of direct traffic at the transport protocol level in the WiMax-1/2 cluster oriented to offload the smart city's wireless ecosystem

**DOI:** 10.1098/rsos.240206

**Published:** 2024-07-03

**Authors:** Viacheslav Kovtun, Krzysztof Grochla, Saad Aldosary, Mohammed Al-Maitah

**Affiliations:** ^1^ Computer Control Systems Department, Faculty of Intelligent Information Technologies and Automation, Vinnytsia National Technical University, Khmelnitske Shose str., 95, Vinnytsia 21000, Ukraine; ^2^ Internet of Things Group, Institute of Theoretical and Applied Informatics Polish Academy of Sciences, Bałtycka 5, 44-100 Gliwice, Poland; ^3^ Computer Science Department, Community College, King Saud University, 11451, Riyadh, Saudi Arabia

**Keywords:** smart city, unlicensed frequency band, WiMax-1/2 cluster, service duration, information transfer, communication resources management

## Abstract

Rerouting of direct information traffic under the WiMax-1/2 technology control in the case of licensed frequency spectrum overload ensures communication continuity in the smart city's critical infrastructure. The support of such a process in the WiMax-1/2 cluster has its specificity, worthy of analytical formalization. The article presents a mathematical apparatus that allows the average service duration of an information message during its transfer from the terminal to the WiMax-1/2 base station to be estimated. Unlike analogues, the presented concept adequately describes the investigated process for any number of terminals, taking into account both the queuing effect on their side and the functioning of the cumulative query transmission mechanism inherent in WiMax-1/2 technology. Therefore, the proposed mathematical apparatus, describing the process of servicing an information message, takes into account both the average duration accompanied by potential collisions in the process of sending a request for the allocation of communication resources for its transmission to the base station, and the average duration of the information message's stay in the terminal queue. Experimental studies demonstrated the adequacy of the proposed mathematical apparatus for describing the investigated process. The experimental section also formulates the optimization problem of the investigated process resulting from the management of competitive access parameters.

## 1. Introduction and state of the art

### 1.1. Relevance of the research

The smart city's infosphere continuously puts a strain on the communication resources that support it around the clock. There are specific time intervals and geographical locations where the communication technologies of the smart city experience overload, resulting in a decrease in the quality of service for subscribers, up to the loss of traffic. Naturally, such a situation is unacceptable when it comes to critical infrastructure traffic in the smart city. For such emergencies, it is necessary to have a backup communication infrastructure. It should be taken into account that the licensed frequency range in urban conditions is saturated with signals. Thus, the nominee for the sought-after backup communication technology, on the one hand, should be standardized to ensure the reliability and security of information traffic and have interfaces for integration with common modern communication technologies. On the other hand, it should provide the ability to use unlicensed frequency ranges in emergencies. According to the authors, WiMAX technology fully meets these criteria.

The WiMAX (Worldwide Interoperability for Microwave Access) telecommunication technology [1–4] was developed to facilitate universal wireless communication over long distances for a wide span of end devices (terminals). This technology is defined by the family of IEEE 802.16 standards [3,5] and represents a broadband wireless access technology designed for implementation in distributed wireless networks at the city-regional level. WiMAX serves as an alternative to cable networks, competing with current mobile standards, and is positioned as a flexible solution for the backbone network of Wi-Fi access points in urban conditions.

The IEEE 802.16 standard outlines the principles for constructing networks on a regional scale in the frequency range of 1.5–11 GHz, where line-of-sight between the receiver and transmitter is not required. More precisely, it outlines the radio interface based on a common Medium Access Control (MAC) protocol, compatible with various Physical Layer (PHY) specifications. These PHY specifications are determined based on the frequency range and licensing constraints, allowing for flexibility in adapting to different scenarios.

Among the specific features of WiMAX that need to be considered in its simulation modelling in a general sense, the following can be noted:

— Orthogonal Frequency Division Multiple Access (OFDM) modulation scheme. This scheme enables the transmission of diverse signals simultaneously using various subcarriers.
— Adaptive Modulation and Coding mechanism. This mechanism adjusts modulation and coding based on channel conditions and interference, ensuring the most efficient use of bandwidth.
— Frequency (FDD, Frequency Division Duplex) and Time (TDD, Time Division Duplex) Multiplexing. These techniques allow the adaptation of the system to the requirements of different countries and regions, providing flexibility in communication.

When simulating WiMAX, taking into account these features is essential for accurately representing the behaviour and performance of the technology in various scenarios.

The technological features of WiMax-1/2 naturally influence the formation of a specialized Quality of Service (QoS) mechanism. WiMax-1/2 employs a mechanism centred on establishing a dedicated link between the base station and the target terminal. Each connection is established using a dedicated scheduling algorithm designed to ensure QoS parameters for individual connections. By contrast, Wi-Fi, for example, uses a QoS mechanism similar to that employed in Ethernet, where packets are assigned different priorities and quality of service guarantees. Therefore, studying the process of transmitting information messages from the terminal to the base station in the conditions of a saturated WiMax-1/2 ecosystem is a relevant scientific task with practical significance.

### 1.2. State of the art

This section explores existing literature on performance analyses under varied assumptions and environments within WiMAX networks [6,7]. The studies aim to enhance performance in specific areas, encompassing analyses of queueing states in resource control, polling assessments under diverse arrival rates and buffer sizes and contention analyses in multicast mode. Detailed explanations of these analyses are provided below.

Initially, regarding the analysis of queueing states in resource control, various studies [8–10] have introduced distinct throughput control schemes. Many of these schemes incorporate the water-filling method as a connection admission control (CAC) but fall short of addressing the polling mode used between the base station and terminals. Additionally, [11,12] presented a CAC that takes into account both available throughput and delay simultaneously. However, as this approach focuses solely on a single terminal within a base station, the analysis outcomes may not apply to real-world networks.

Secondly, concerning polling analysis, [13,14] formulated an analytical model to assess the average latency of a data packet in a scenario where nodes are polled sequentially after each uplink subinterval. They further presumed that the base station can poll all terminals within a single frame period. However, this assumption becomes impractical when the frame length is finite, potentially exceeding the available slots for polling control.

Thirdly, numerous studies [8,15–17] have delved into the contention period for registration in the ‘initial ranging period' and the resource request for multicast/broadcast during the ‘resource request period'. [8,16] concluded that WiMAX achieves maximum throughput when a contention window size equals the number of competitive users' terminals. However, in a distributed network, the number of competitive terminals is unknown beforehand. Specifically, not all served terminals have packets to transmit at any given time. Consequently, the base station is aware of the total number of terminals but lacks information about how many terminals wish to be served. [17] examined the impact of overheads in MAP (Media Access Control) messages, highlighting their contribution to spectral inefficiency for VoIP (Voice over IP) services in the WiMAX OFDMA system.

By formulating a throughput-over-utilization cost function, [18,19] conducted an analysis determining the contention period's optimal size to be (2*M* − 1), where *M* represents the competitive terminals' number. The challenge identified in [18] mirrors that in [20], where the number of competitive terminals remains unknown during a random contention procedure. [21] also introduced a weighted factor, for each metric related to utilization, throughput and dropping probability to derive the initial backoff window optimal size [22] utilizing a Markov chain model, investigated throughput under varying contention window sizes and terminals' numbers. In [23] the mean latency with a constant contention window size was examined. Concurrently, [24] created a cross-layer analytical framework to design the contention period optimal size. It is worth noting that [18–26] all assumed a constant contention window size in their analyses. [26] introduced an analytical framework that computes data transmission latency, contention latency and throughput, considering varied assignments of the contention period. The proposed adaptive contention slots allocation mechanism enables the attainment of optimal utilization. However, it's crucial to highlight that the analytical results may not align with the requirements of real WiMAX networks, as they don't account for varying numbers of polls during a frame interval [27]. Additionally, [28] introduced a sleep mode for WiMAX, aiming to reduce energy consumption and average latency.

Extensive studies have been conducted on Wi-Fi offloading due to the advantages of diverting mobile network data to Wi-Fi. These benefits primarily include reducing base station power consumption, alleviating network congestion and enhancing quality of service for users, particularly in densely populated areas. Research in this field began over ten years ago, but owing to its significant benefits and ongoing network advancements, it remains an active area of investigation, with Wi-Fi offloading also being considered for 5G networks. Reference [29] introduced a bid-based heterogeneous resource allocation framework that allows mobile network operators to simultaneously and efficiently utilize both cellular and operator-owned Wi-Fi resources. This framework employs an auction-based mechanism to facilitate dynamic Wi-Fi offloading, taking into account user valuations. Operator-domain offloading prevents selfish user behaviour and ensures near-optimal profitability and social utility. Reference [30] presented a method for joint Wi-Fi and cellular offloading aimed at optimally reducing energy consumption and latency in mobile terminals during task processing.

Reference [31] introduced a Wi-Fi offloading algorithm that takes traffic congestion awareness into account. The algorithm considers user traffic information based on usage patterns (e.g. video streaming or emails), effectively assigning a user priority level. Users with higher priority levels are given precedence for Wi-Fi offloading. However, the study does not address the signalling overhead required to update the priority level information, which likely fluctuates over time. Reference [32] suggested an incentive mechanism to motivate Wi-Fi access point owners to engage in data offloading. Their approach, the Delay-constraint and Reverse Auction-based Incentive Mechanism, addresses an optimization problem aimed at maximizing the mobile network operator's revenue while also considering the delay constraints of various user applications. Additionally, they proposed a Vickrey–Clarke–Groves scheme-based payment rule to ensure individual rationality and truthfulness among users, accounting for different traffic load scenarios. Reference [33] proposed an optimal pricing strategy to enhance user satisfaction through Wi-Fi offloading and reduce the financial losses for mobile network operators caused by users offloading to Wi-Fi.

To summarize, the majority of studies have focused on determining the contention window optimal size for initial ranging and throughput contention in group polling, with limited attention given to unicast polling. Moreover, many studies have not concurrently addressed critical factors such as the number of terminals, number of polls and the lengths of data slots and control slots in the uplink/downlink subintervals. As a result, the findings apply only to specific areas within WiMAX and may not provide comprehensive insights across the entire network.

### 1.3. Main attributes of the research

The ***object*** of the research is a process of servicing an information message within the WiMax-1/2 cluster, starting from the moment it enters the terminal queue until the successful completion of its transfer to the base station.

The ***subject*** of the research encompasses the teletraffic theory, queueing theory, Markov chain theory and methods of functional analysis.

The ***aim*** of the research is to evaluate the average duration of servicing an information message transmitted from the terminal to the base station within the WiMax-1/2 cluster, taking into account the specific characteristics of the operation of the underlying transport protocol.

Research ***tasks***:

— Define the parameter space in a formalized manner to ensure a comprehensive representation of the research object (§2.1).
— Formalize the concept of evaluating the average duration of the process of sending a request for the allocation of communication resources to transmit an informational message, taking into account potential collisions (§2.2).
— Formalize the concept of evaluating the average duration of transmitting an informational message from the terminal queue to the base station's information environment, considering the characteristics of WiMax-1/2 technology and the cumulative mechanism of request transmission (§2.2).
— Estimate the average duration an informational message stays in the terminal queue (§2.2).
— Conduct a numerical experiment to validate the efficacy of the developed mathematical apparatus (§3).

In summary, let's formulate a ***main contribution*** of the research. The article presents a mathematical apparatus that allows estimating the average service duration of an information message during its transfer from the terminal to the WiMax-1/2 base station. Unlike analogues, the presented concept adequately describes the investigated process for any number of terminals, taking into account both the queuing effect on their side and the functioning of the cumulative query transmission mechanism inherent in WiMax-1/2 technology. Therefore, the proposed mathematical apparatus, describing the process of servicing an information message, takes into account both the average duration accompanied by potential collisions in the process of sending a request for the allocation of communication resources for its transmission to the base station and the average duration of the information message's stay in the terminal queue. The experimental section also formulates the optimization problem of the investigated process resulting from the management of competitive access parameters.

## 2. Material and methods

### 2.1. Statement of the research

Let's consider a centralized cluster of a WiMax-1/2 wireless network operating in TDD mode with orthogonal frequency-division channel multiplexing. The infrastructure of the network cluster includes a WiMax-1/2 base station and *K* terminals. The investigated network operates in discrete time with an elementary interval of duration *D*. To handle bidirectional traffic within any elementary interval *D*, two sub-intervals *D*_DF_, *D*_DF_ are allocated, such that *D* = *D*_DF_ + *D*_BF_. During the *D*_DF_ sub-interval in the cluster, servicing of the direct information traffic (from terminals to the base station) takes place. Similarly, during the *D*_BF_ sub-interval in the cluster, servicing of the backward information traffic (from the base station to terminals) occurs. The transfer within the *D*_BF_ sub-interval begins with the transmission of control information in the form of a schedule. This schedule regulates, for each *k*-th terminal, *k* ∈ *K*, the frame when this terminal will be allowed for the forward information transfer within the next *D*_DF_ sub-interval.

Information regarding the necessity of Allocating Communication Resources (ACR) is directed by terminals to the base station in the form of target ACR requests during the *D*_DF_ sub-interval. The base station responds to ACR requests by allocating target frames with a total quantity *F* in the distribution scheme for the next *D*_BF_ sub-interval, taking into account the current volumes of buffered direct and backward traffic. It's important to note that a terminal can send an ACR request to the base station either by using specially designated frames (allocated by the base station as a result of polling target terminals) or by attaching the request to the already expected data sent to the base station. Additionally, terminals can send ACR requests to the base station during a competitive access frame with a finite number of slots *N*, which serves as the concluding element of each sub-interval *D*_DF_.

The multiple interaction of terminals when sending ACR requests to the base station during the default competitive access frame is controlled by the BEB (Binary Exponential Backoff) algorithm. According to the BEB algorithm, a terminal sends ACR requests at time intervals equal to *τ*. The value of *τ* for the *i*-th attempt to send an ACR request is chosen from the interval $\left(0,{\hat{S}}_{i}-1\right)$, where ${\hat{S}}_{i}$ is the current value of the Collision Resolution Window. If the inequality *y* = tranc(*τ*/*N*) > 0 holds for the integer part *y* of *τ*/*N*, the terminal skips *y* frames and sends the ACR request in the *τ* − *yN*-th slot of the -th frame. The terminal recognizes the loss of an ACR request if, during the next *D*_rt_ frames after its transmission, the base station does not allocate target communication resources for data transmission. In the context of WiMax-1/2 technology, the value of the constant *D*_rt_ is determined by the base station for the cluster. In the event of a recognized loss of an ACR request, the terminal retries sending it with the doubling of CRW if the value of the latter has not yet reached the maximum ${\hat{S}}_{\max}=2^{m}{\hat{S}}_{\min}=2^{m}NS_{0}$, where $S_{0}\in N$ is the minimum CRW, and the multiple of *N* contributes to reducing the probability of collisions in the implementation of competitive access: ${\hat{S}}_{i}=NS_{0}$, $i=\bar{0,m}$.

Taking into account the TDD protocol, the process of transmitting data from the terminal to the base station begins with a preamble of one OFDM symbol duration, followed by several Medium Access Control (MAC) layer data packets represented by a corresponding number of OFDM symbols. Moving forward, let's assume that the informational message (data sent by the terminal to the base station) contains one MAC layer packet, which is composed of a specific number of OFDM symbols.

The purpose of this study is to assess the service duration of an informational message transmitted from the terminal to the base station in a WiMax-1/2 cluster. The servicing process commences from the moment the information message enters the transmission queue on the terminal side and continues until the moment of receipt of the last confirmation of the receipt of this message by the base station. The functioning of a WiMax-1/2 network specifies that the base station, at the beginning of the *D_i_*, *i* > 0, i∈N, interval, sends messages to the corresponding terminals regarding the successful receipt of informational messages sent to it during the *D_i_*_−1_ interval. Taking this into consideration, the moment of completion of servicing the informational message will be considered the moment at the end of the *D_i_*_−1_ interval, provided that the terminal receives a confirmation message at the beginning of the *D_i_* interval.

If the base station receives an ACR request from a target terminal during the *D_j_*, *j* < *i*, j∈N, interval, it reserves communication resources for the transfer of the corresponding informational message during the subsequent *D_i_*_−_*_j_*_−1_ intervals. In this context, let's introduce a parameter *D*_rg_, which represents the time from the moment the informational message enters the transmission queue on the target terminal side to the completion of the *D_j_* interval during which the base station received the corresponding ACR request.

Additionally, let's introduce a parameter *D*_sd_ that represents the time from the moment *D*_rg_ until the completion of the interval *D_i_*_−1_, during which the base station successfully receives the corresponding information message from the target terminal. The mentioned duration of servicing the information message in the previous paragraph is determined as the sum of parameters *D*_rg_ and *D*_sd_.

Let's further formalize the model of direct information traffic in the WiMax-1/2 cluster, based on the models for estimating the mean values of parameters *D*_rg_ and *D*_sd_. In doing so, we will specify a series of assumptions:

— On any *k*-th terminal, *k* ∈ *K*, the queue of information messages ready for transmission is replenished with intensity *η* in accordance with the Poisson low.
— We will assume that the information transmission environment does not introduce interference to the traffic in the WiMax-1/2 cluster.
— The traffic rate is higher than the overall intensity of arrival of ready-to-send information messages on the side of terminals, i.e. the inequality *KηD* < *F* holds.
— If the base station received an ACR request from the terminal during the interval *D_i_*, it will allocate the corresponding communication resources to serve the target traffic in one of the subsequent $D_{i+D_{rt}}$ intervals.

### 2.2. The model of direct information traffic in a WiMax-1/2 cluster

Let's represent the WiMax-1/2 cluster as a queuing system with an input flow of ACR requests with an intensity of *Kη*. This intensity characterizes the dynamics of the average number of information messages whose processing is authorized by the base station. The state of such a cluster at the moment *t* is described by the function of the number of registered information messages *i*(*t*). Let's investigate such a queuing system in discrete time, where the time quantum is equal to the duration of the interval *D*.

At the moment *t_w_*, the completion of the *w*-th interval causes a step change in the number of registered information messages in the cluster, as a portion of them finishes processing. Additionally, at this moment, the servicing of *F* new information messages is initiated, which will conclude at the end of the *w* + 1-th interval after the target terminals receive the corresponding messages from the base station. Generalizing the logic of this process, we can state: if at the moment *t_w_* the cluster had *i* ≥ *F* registered information messages, then, for the system to have *j* registered information messages at the moment *t_w_*_+1_, it is necessary that during the (*t_w_*, *t_w_*_+1_) interval *j* − *i* + *F* information messages arrive at the cluster.

If the system *i*(*t_w_*) registered number of information messages is equal to *i* < *F*, the servicing of all these information messages will take place within an interval of (*t_w_*, *t_w_*_+1_). A new information message will have to wait in the queue at the beginning of the *t_w_*_+1_-th interval. In the context of the above, let's define the positive transition matrix elements for the process *i*(*t_w_*) as

$$\left\{ \begin{array}{c} \pi_{i,j}=l_{j}\forall 0\leq i<F,\;\\ \pi_{i,j}=l_{\;j-i+F}\forall i\geq F,j\geq i-F,\; \end{array}\right.$$

where *l_n_* is the probability of receiving *n* new information messages to the investigated cluster during any *D* interval. Certainly, let's formulate the system of equilibrium equations and normalization equations as follows

2.1 $$\begin{array}{rl} q_{j} & =l_{j}\sum_{i=0}^{F-1}q_{i}+\sum_{i=F}^{F+j}q_{i}l_{\;j-i+F},j\geq 0,\\ \sum_{\;j=0}^{\infty }q_{j} & =1, \end{array}$$

where *q_j_* is the stationary probability that a cluster contains *j* informational messages.

To simplify the solution process of system (1), we will disregard *q_j_* if *j* > *M*, where *M* is a sufficiently large number. Solving a system of the form (2.1), consisting of equations concerning variables $q=(q_{0},\ldots q_{M})$, can be achieved, for example, using the Gaussian method [34]. With the determined vector ***q*** , we define the average total number of registered informational messages in a cluster as

2.2 $$L=\sum_{\;j=0}^{M}\;jq_{j}.$$

Based on the expression (2.2), the average time *D*_sd_ can be expressed using Little's formula as

2.3 $$D_{sd}=\frac{L}{\left(K\eta \right)}.$$

Now, let's focus on formalizing the metric *D*_rg_. In §2.1, we emphasized that the terminal waits for the allocation of channel resources to transmit an information message during the time *D*_rt_ from the moment of sending the ACR request. If the time equal to *D*_rt_ elapses and the base station has not allocated channel resources, the terminal considers the previously sent ACR request lost and retransmits it. In this context, the state in which the terminal is at the beginning of the *t*-th interval can be defined in the space of two characteristics:

— The current *v*(*t*) = (0, … , *m*) iteration in attempting to send an ACR request to the base station. In turn, the function *v*(*t*) is characterized by the current value of CRW *S_i_*, which corresponds to the number of attempts *i* < *m* to send an ACR request.
— The parameter *u*(*t*), the value of which, at *u*(*t*) < 0, corresponds to the value of *D*_rt_, and at *u*(*t*) ≥ 0 equals the number of *D* intervals remaining until the next attempt to send an ACR request.

The start of the *v*(*t*) iteration is defined as the beginning of the interval *D* that occurs after the terminal's futile waiting *D*_rt_ for a message allocating channel resources in response to a previously sent ACR request. We consider the zeroth iteration *v*(0) as the moment when the terminal appears in the queue without allocated channel resources, and there is an informational message that needs to be sent to the base station. The end of the *v*(*i*), *i* ∈ (1, *m* − 1), iteration is considered the beginning of the interval *D* during which channel resources are allocated to the terminal for transmitting the informational message.

After receiving a message confirming the successful reception of the ACR request by the base station, the terminal transitions either to the state of the zeroth iteration *v*(0) (if a new informational message appeared in its queue during the next interval *D*) or to the waiting state *C* (when registering an informational message in the queue, the terminal transitions from the state *C* to the state of the zeroth iteration *v*(0)).

In summary, let's characterize the behaviour of the terminal in the WiMax-1/2 cluster using a two-dimensional discrete stochastic process {v(t)=i,u(t)=n}∪{C}, where the time quantum is equal to the interval *D*. The transition probabilities of such a process, represented by a Markov chain, can be described by the following expressions:

$$Q\{0,n|C\}=\frac{Q_{0}}{S_{0}\forall n\in (0,S_{0}-1)},$$

$$Q\{i,n|i,n+1\}1\forall n\in (-D_{rt},S_{i}-2),n\neq -1,i\in (0,m),$$

$$Q\{C|i,0\}=(1-q_{c})(1-Q_{0})\forall i\in (0,m),$$

$$Q\{0,n|i,0\}=\frac{Q_{0}(1-q_{c})}{S_{0}}\;\forall n\in (0,S_{0}-1),i\in (0,m),$$

$$Q\{i,-1|i,0\}=q_{c}\forall i\in (0,m),$$

$$Q\{i,n|i-1,-D_{rt}\}=\frac{1}{S_{i}}\forall n\in (0,S_{i}-1),i\in (0,m-1)$$

$$\mathrm{and}\;\;\;\;\;\;Q\{m,n|m,-D_{rt}\}=\frac{1}{S_{m}}\forall n\in (0,S_{m}-1),$$

where *q*_c_ is the probability of collision occurrence during the transmission process of the ACR request, and *Q*_0_ = 1 − exp( − *η*) is the probability that at least one informational message will appear in the terminal within the interval *D*.

Let's characterize the state (*i*, *n*) of the process {v(t)=i,u(t)=n}∪{C} with the stationary probability *τ_i_*_,*n*_, where

2.4 $$\tau_{i,n}=\tau_{i,n-1}+\tau_{i,S_{i-1}}\;\forall n\in (0,S_{i}-2),i\in (0,m).$$

Based on the expression (2.4) for the zeroth iteration *v*(0), let's write:

2.5 $$\tau_{0,n}=Q_{0}\left(Q\{C\}+(1+q_{c})\sum_{i=0}^{m}\tau_{i,0}\right)\frac{S_{0}-n}{S_{0}}\forall n\in (0,S_{0}-1)$$

and

2.6 $$\tau_{0,n}=q_{c}\tau_{0,0}\forall n\in (-D_{rt},-1),$$

and for the *i*-th iteration, *i* ∈ (1, *m* − 1), let's write:

2.7 $$\tau_{i,n}=q_{c}\frac{(S_{i}-n)\tau_{i-1,0}}{S_{i}}\forall n\in (0,S_{i-1}),i\in (0,m-1)$$

and

2.8 $$\tau_{i,n}=q_{c}\tau_{i,0}\forall n\in (-D_{rt},-1),i\in (0,m-1).$$

On the *m*-th iteration, the value of CRW reaches its maximum at ${\hat{S}}_{\max}$ and does not increase further, i.e.:

2.9 $$\tau_{m,n}=\frac{q_{c}(S_{m}-n)\tau_{m-1,0}}{(1-q_{c})S_{m}}\forall n\in (0,S_{m--1})$$

and

2.10 $$\tau_{m,n}=q_{c}\tau_{m,0}\forall n\in (-D_{rt},-1).$$

Based on the expressions (2.4)–(2.10), we express the stationary probabilities of states {*τ*_0,*n*_, *τ_i_*_,*n*_, *τ_m_*_,*n*_} in terms of *τ*_0,0_:

2.11 $$\tau_{0,n}=Q_{0}(Q\{C\}+\tau_{0,0})\frac{S_{0}-n}{S_{0}}\forall n\in (0,S_{0}-1),$$

2.12 $$\tau_{0,n}=q_{c}\tau_{0,0},\;n\in (-D_{rt},-1),$$

2.13 $$\tau_{i,n}=q_{c}^{i}\frac{(S_{i}-n)\tau_{0,0}}{S_{i}}\forall n\in (0,S_{i}-1),i\in (0,m-1),$$

2.14 $$\tau_{i,n}=q_{c}^{i+1}\tau_{0,0}\forall n\in (-D_{rt},-1),i\in (0,m-1),$$

2.15 $$\tau_{m,n}=q_{c}^{m}\frac{(S_{m}-n)\tau_{0,0}}{(1-q_{c})S_{m}}\forall n\in (0,S_{m}-1)$$

2.16 $$\mathrm{and}\;\;\;\;\;\;\;\tau_{m,n}=q_{c}^{m+1}\frac{\tau_{0,0}}{1-q_{c}}\forall n\in (-D_{rt},-1).$$

Now we need to formalize the normalization condition for expressions (2.11)–(2,16). Let's express the probability *Q*{*I*} from expression (2.11) in terms of *τ*_0,0_ at *n* = 0:

2.17 $$Q(C)=\frac{1-Q_{0}}{Q_{0}}\tau_{0,0}.$$

Based on expression (2.17), we analytically formalize the desired normalization condition for expressions (2.11)–(2.16):

2.18 $$Q\{C\}+\sum_{i=0}^{m}\sum_{n=-D_{rt}}^{S_{i-1}}\tau_{i,n}=1.$$

After substituting (2.18) into the system (2.11)–(2.16) and the corresponding transformations, we obtain:

$$\tau_{0,0}=(\frac{1-Q_{0}}{Q_{0}}{+\frac{\left(1-2q_{c})(1+S_{0}+2q_{c}D_{rt})+q_{c}S_{0}(1-{\left(2q_{c}\right)}^{m}\right)}{2(1-q_{c})(1-2q_{c})})}^{-1}.$$

Let's now determine the probability υ that the terminal sends an ACR request in any randomly chosen slot within the given interval *D*. The ACR request is sent when *u*(*t*) = 0 is irrespective of the iteration number , *i* ∈ (0, *m* − 1), so the probability of choosing a specific interval *D* for sending the ACR request can be characterized by the expression

2.19 $$\upsilon_{D}=\sum_{i=0}^{m}\tau_{i,0}=\frac{\tau_{0,0}}{1-q_{c}}.$$

As the slots for sending ACR requests within the interval *D* are uniformly distributed, and their total number equals *N*, the sought probability υ of choosing any slot within the interval *D* can be described by the expression

2.20 $$\upsilon =\frac{1}{N}\sum_{i=0}^{m}\tau_{i,0}=\frac{\tau_{0,0}}{N(1-q_{c})}.$$

Collision during the transmission of an ACR request occurs if at least one of the remaining *K* − 1 terminals also chooses the same slot for sending an ACR request. In the context of expressions (19) and (20), we will define the probability of collision *q_c_* during the transmission of an ACR request as

2.21 $$q_{c}=1-{\left(1-\upsilon \right)}^{K-1}=1-{\left(1-\frac{\tau_{0,0}}{N(1-q_{c})}\right)}^{K-1}.$$

As a result, to find the two unknown parameters *τ*_0,0_ and *q*_c_, we have a system of two equations (2.4) and (2.21). A potentially permissible simplification of this system is the assumption that *Q*_0_ = 1 is equal to *D_rt_* = 0.

Now, let's formalize the estimation of the average time *D*_rg_ (see §2.1). By definition, the value of parameter *D*_rg_ is directly proportional to the mathematical expectation E[*h*] of the total registration time for informational messages, and is inversely proportional to the mathematical expectation E[*k*] of the number *k* of such informational messages. The registration of an informational message by the base station is directly related to the effectiveness of the ACR request transmission process. In this context, let's divide the ACR request transmission time into two periods. The first period begins from the moment an informational message enters the terminal's queue (hereafter referred to as the first information message) where there were no unregistered informational messages before, and lasts until the beginning of the next interval *D*. The second period starts from the completion of the first period and continues until the transfer of the first informational message is completed.

Let's consider the case where the duration of the first period is *y* ∈ (0, 1], and the duration of the second period is *j* of *D* intervals. The probability of realizing such a case can be estimated as (1 − exp( − *η*))^−1^*q_j_η*exp( − *ηy*)*dy*, where *q_j_* is the probability that the duration of the second period is *j* of *D* intervals. When sending an ACR request for the first information message to the terminal queue, new information messages may arrive. Let's estimate their average quantity by the value *η*(*y* + *j*) and the average total duration of their registration by the value *η*(*y* + *j*)^2^/2. Taking into account the first information message, we can write:

2.22 $$D_{rg}=\frac{E[h(y,j)]}{E[k(y,j)]},$$

where *h*(*y*, *j*) = *y* + *j* + *μ*(*y* + *j*)^2^/2, *k*(*y*, *j*) = 1 + *η*(*y* + *j*).

For practical application of relation (2.22), it is necessary to estimate the probability *q_j_*. To do this, let's first determine the distribution of the number of intervals *D* during which an ACR request was sent, given that the terminal successfully transmitted this request on the *z*-th attempt. On the *i*-th attempt to send an ACR request, the terminal, to do this, equally likely chooses one of *S_i_* slots, 0 < *i* < *z*. Let's express the generating function of the duration of this attempt given 0 < *i* < *z*:$\Omega_{i}(a)=(1/S_{i})\sum_{\;j=1}^{S_{i}}a^{\;j+D_{rt}}$. For any given value of *i*, the generating function s will take the form

$$\Omega_{i}(a)=\left\{ \begin{array}{c} \frac{1}{S_{i-1}}\sum_{\;j=1}^{S_{i-1}}a^{\;j+D_{rt}}\forall i=\bar{1,m+1},\;\\ \Omega_{m+1}(a)\forall i>m+1.\; \end{array}\right.$$

During the first attempt, the first interval *D* is fractional, accordingly: $\Omega_{1}(a)a^{-1}=1/S_{0}\sum_{\;j=0}^{S_{0}-1}a^{\;j+D_{tr}}$. On the *z*-th attempt, the terminal waits for a duration of *D_rt_*, so the generating function can be expressed as $\Omega_{z}(a)a^{-D_{rt}}$. The duration of each attempt to send an ACR request is an independent stochastic variable. Therefore, in terms of the generating function, the duration of the process of sending an ACR request, which was successfully realized on the *z*-th attempt, can be represented as $\Psi_{z}(a)=a^{-D_{rt}-1}\prod_{i=1}^{z}\Omega_{i}(a)$ measured in whole intervals *D*. Taking into account the probabilities of successful transmission of the ACR request on different attempts, we obtain the generalized form of the generating function Ψ(*a*):

2.23 $$\Psi (a)=(1-q_{c})\sum_{z=0}^{\infty }q_{c}^{z}\Psi_{z}(a)=(1-q_{c})a^{-D_{rt}-1}\left(\sum_{z=0}^{m}q_{c}^{z}\prod_{i=1}^{z}\Omega_{i}(a)+\frac{q_{c}^{m}}{1-q_{c}\Omega_{m+1}(a)}\prod_{i=1}^{m+1}\Omega_{i}(a)\right)$$

From equation (2.23), we can express the desired probabilities *q_j_* as $q_{j}=1/\;j!\;(d^{j}\Psi (a)/da^{j}){|}_{a=0}$. This allows us to analytically formalize the mathematical expectations E[*h*] and E[*k*] as

2.24 $$\begin{array}{rl} E[h(y,j)]= & \sum_{\;j=0}^{\infty }\left(\frac{\eta q_{j}}{1-\exp(-\eta )}\int_{0}^{1}\left(y+j+\frac{\eta {\left(y+j\right)}^{2}}{2}\right)\exp(-\eta y)\;dy\right)\\ & =\frac{2}{\eta }-\frac{\exp(-\eta )}{1-\exp(-\eta )}\left(\frac{\eta }{2}+2\right)+\left(2-\frac{\eta \exp(-\eta )}{1-\exp(-\eta )}\right)\sum_{\;j=0}^{\infty }\;jq_{j}+\frac{\eta }{2}\sum_{\;j=0}^{\infty }\;j^{2}q_{j}, \end{array}$$

and

2.25 $$\begin{array}{rl} E[k(y,j)]= & \sum_{\;j=0}^{\infty }\left(\frac{\eta q_{j}}{1-\exp(-\eta )}\int_{0}^{1}(1+\eta (y+j))\exp(-\eta y)\;dy\right)\\ & =2-\eta \frac{\exp(-\eta )}{1-\exp(-\eta )}+\eta \sum_{\;j=0}^{\infty }\;jq_{j}. \end{array}$$

Let's transform the expressions (2.24) and (2.25) taking into account the known property of the generating function $\sum_{\;j=0}^{\infty }\;jq_{j}=\Psi^{\prime }(1)$ and the form of the derivative $d\Omega_{i}/da{|}_{a=1}=D_{rt}+(S_{i-1}+1/2)$. As a result, we get:

2.26 $$E[h(y,j)]=\frac{2}{\eta }-\frac{\exp(-\eta )}{1-\exp(-\eta )}\left(\frac{\eta }{2}+2\right)+\left(2-\frac{\eta \exp(-\eta )}{1-\exp(-\eta )}+\frac{\eta }{2}\right)\Psi^{\prime }(1)$$

and

2.27 $$E[k(y,j)]=2-\frac{\eta \exp(-\eta )}{1-\exp(-\eta )}+\eta \Psi^{\prime }(1),$$

where $\Psi^{\prime }(1)=-1+(1/2)\sum_{i=1}^{m-1}(S_{i}+1)q_{c}^{i}+D_{rt}q_{c}/1-q_{c}+(q_{c}^{m}(S_{m}+1)/2(1-q_{c}))$.

Substituting expressions (2.27) and (2.26) into expression (2.22) allows us to estimate the parameter *D*_rg_.

Finally, let's estimate the probability of new information messages arriving *l_n_* at the investigated cluster during any interval *D*. This estimate is necessary for calculating the average time *D*_sd_ (see §2.1, equations (2.1), (2.3)).

Let's determine the distribution {*ϕ_n_*} of the number of information messages, for the service of which communication resources were ordered in the successfully sent given ACR request. The distribution of the quantity of informational messages arriving at the terminal queue during the first period (including the first information message) can be interpreted as the conditional distribution of the quantity of received informational messages (assuming that at least one was successfully received):

2.28 $$\phi_{0}(n+1)=\frac{\left(\eta^{n}\exp(-\eta )\right)}{\;/\left(n!(1-\exp(\eta ))\right)\forall n}=0,1,\ldots .$$

The generating function of the distribution (2.28) has the form

2.29 $$\varphi_{0}(a)=\frac{\left(a\exp(-\eta +\eta a)\right)}{\left(1-\exp(-\eta )\right)}.$$

Let's determine the generating function of the quantity of informational messages that were received during the transmission of the ACR request (excluding the first period). The mentioned quantity can be interpreted as the sum of independent stochastic variables, each representing the quantity of informational messages received during a consecutive interval *D*. These stochastic variables have the same distribution. The number of terms in the sum is also random and is characterized by the generating function Ψ(*a*). Consequently, the generating function of the quantity of informational messages received during a consecutive interval *D* is defined as exp( − *η* + *ηa*). Therefore, the generating function of such a sum looks like this Ψ(exp( − *η* + *ηa*)). Based on this, we can express it as: φ(*a*) = φ_0_(*a*)Ψ(exp( − *η* + *ηa*)).

Let's formalize the distribution of the number of terminals that successfully transmitted ACR requests during any arbitrarily chosen interval *D*. The probability that a specific terminal selected this interval *D* for transmitting an ACR request is equal to υ*_D_* (see expression (2.19)). Terminals independently choose slots for sending ACR requests. This allows us to determine the probability that *z* terminals of *K* chose the given interval *D*, using the Bernoulli formula: $Q_{tr}(z)=C_{K}^{z}\upsilon_{D}^{z}{\left(1-\upsilon_{D}\right)}^{K-z}$.

Accordingly, the probability that during a given interval *D*, out of *N* slots available for transmitting ACR requests, *z* terminals successfully transmitted *m* ACR requests is determined by the expression

2.30 $$Q_{\sum tr}(m|z)=\sum_{\beta =1}^{](z-m)/2[}C_{N}^{\beta +m}C_{\beta +m}^{m}B(\beta ,z-m),$$

where B(*β*, *n*) is a function representing the number of ways to place *n* ACR requests in *β* slots so that no more than two ACR requests end up in each:

$$B(\beta ,n)=1-\beta^{-n}\left\{\sum_{x=1}^{\beta -1}C_{x}^{\beta }B(x,n)+\sum_{v=1}^{\beta -1}C_{v}^{\beta }\frac{n!}{(n-v)!}\sum_{x=1}^{\beta -1}C_{x}^{\beta -v}B(x,n-v)\right\}.$$

Taking into account expression (2.30), we characterize the probability of observing *m* instances of successful transmission of ACR requests during one interval *D* as

$$Q_{\sum tr}(m)=\sum_{z=m}^{K}Q_{\sum tr}(m|z)Q_{tr}(z).$$

Let's assume that $q_{\sum tr}(v)$ is the generating function for the distribution $\left\{Q_{\sum tr}(m)\right\}$ and the number of information messages, for which communication resource requests are sent in ACR requests by different terminals, being identically distributed independent stochastic variables. In this context, the sought probability *l_n_* of registering *n* information messages in one interval *D* will be defined as

$$l_{n}=\frac{1}{n!}{\frac{d^{n}q_{\sum tr}(\varphi (v))}{dv^{n}}|}_{z=0}.$$

## 3. Results and discussion

Let's ensure the adequacy and functionality of the mathematical apparatus presented in §2 by conducting experimental studies with its involvement.

We investigate the process of direct transmission of information messages in the WiMax-1/2 cluster with the following parameters: minimum value of CRW_*S*_0_ = 2, maximum value of CRW_*S*_max_ = 16, interval duration *D* = 10 ms, duration of OFDM symbol is 12 mcs, and a throughput of the input channel of the base station is 70 Mbps.

With such a throughput, it is possible to transmit an information packet of 96 bytes in one OFDM symbol. Therefore, over *M* OFDM symbols, the terminal can send bytes of data to the base station. The utilization of the communication channel is considered effective if the proportion of control data in the total volume of transmitted information does not exceed 20%. Taking this rule into account for the investigated system, we obtain the value of *M* ≥ 8. It is not difficult to assess that for the investigated system, an information message formed by a sequence of *M* = 8 OFDM symbols has a size of 576 bytes, which corresponds to the typical Maximum Transmission Unit (MTU) size for the TCP/IP protocol. In further experiments, we assumed that the MAC layer block size is 8 OFDM symbols. Accordingly, in the investigated network, the base station, in response to an ACR request, allocated communication resources to the target terminal sufficient for transmitting precisely this number of OFDM symbols.

**Figure 1.**
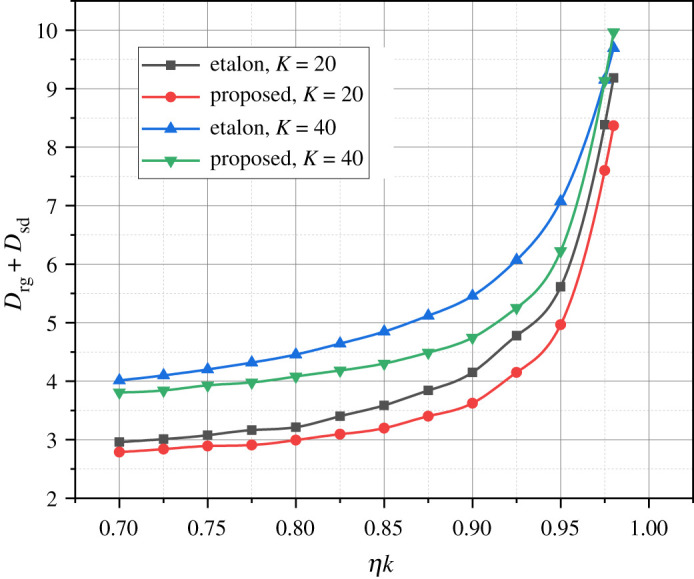
Results of the calculation of variants of the dependence *D*_rg_ + *D*_sd_ = *f*(*η_k_*) at *K* = {20, 40}.

In figure 1, the graphs depict the dependence *D*_rg_ + *D*_sd_ = *f*(*η_k_*) of the total duration of servicing an information message, in the parameterized WiMax-1/2 cluster. The values of parameters *D*_rg_ and *D*_sd_ were calculated using the mathematical apparatus presented in §2, generalized by expressions (2.22) and (2.3), respectively. The abscissa *η_k_* represents the normalized intensity of incoming information messages (sequences of 8 OFDM symbols). Normalization assumes that the parameter *η_k_* represents the fraction of throughput without considering the duration of contention access, i.e. *η_k_* = *KηD*/*F* at *N*, *F* = const (for our experiments, g was used *N* = 36, *F* = 50). The etalon data in figure. 1 are based on the materials of the study [22].

From the graphs presented in figure 1, it can be observed that the discrepancy between the etalon curves and those obtained using the author's mathematical apparatus is small enough to consider the latter adequate.

The interpretation of the nonlinear nature of the graphs presented in figure 1 requires additional investigation, which essentially involves optimizing the functioning process of the investigated network. The mentioned optimization aims to minimize the total duration of servicing an information message, taking into account the specified values of the direct subinterval duration *D*_DF_ and the intensity *η* of the incoming stream of information messages, and varying the relationship between the data transfer duration *F* and the duration of the contention access frame *N*.

In the study, we considered that the subinterval *D_DF_* size was approximately half the size of the elementary interval *D*, i.e. *D_DF_* ≈ 440 OFDM symbols. For the investigated network, the process of sending one ACR request contributes to 2 OFDM symbols, while the process of sending one information message takes 8 OFDM symbols. Therefore, during the mentioned subinterval *D_DF_*, it is possible, for example, to simultaneously transmit 50 information messages (i.e. *F* = 50) and allocate 20 slots for sending ACR requests (i.e. *N* = 20). These values are equivalent to the total throughput of direct information transfer with a speed in the range of 25÷30 Mbps. Assuming that the equality 8*F* + 2*N* = 440 continues to hold, we can manipulate the values of parameters *F* and *N*, thereby influencing qualitative indicators of the functioning of the investigated WiMax-1/2 cluster, such as *D*_rg_ (duration of registration of information message), *D*_sd_ (duration of servicing an information message) and *D*_rg_ + *D*_sd_.

**Figure 2.**
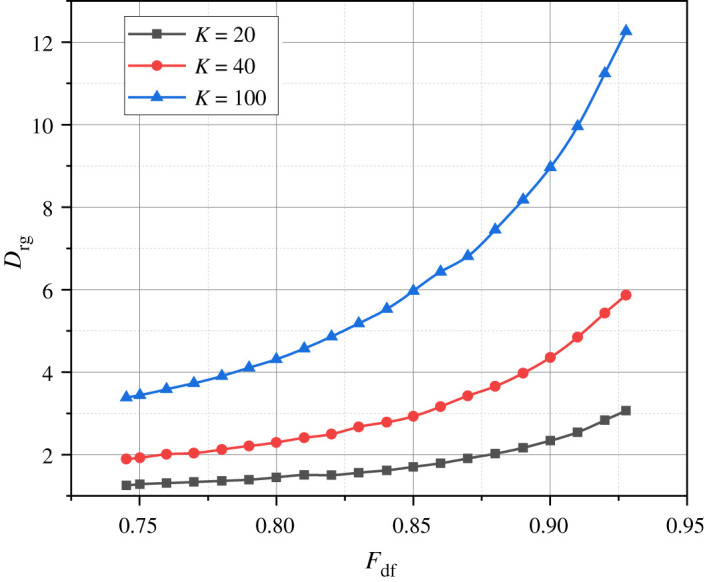
Results of the calculation of variants of the dependence *D*_rg_ = *f*(*F*_DF_) at *K* = {20, 40, 100}.

**Figure 3.**
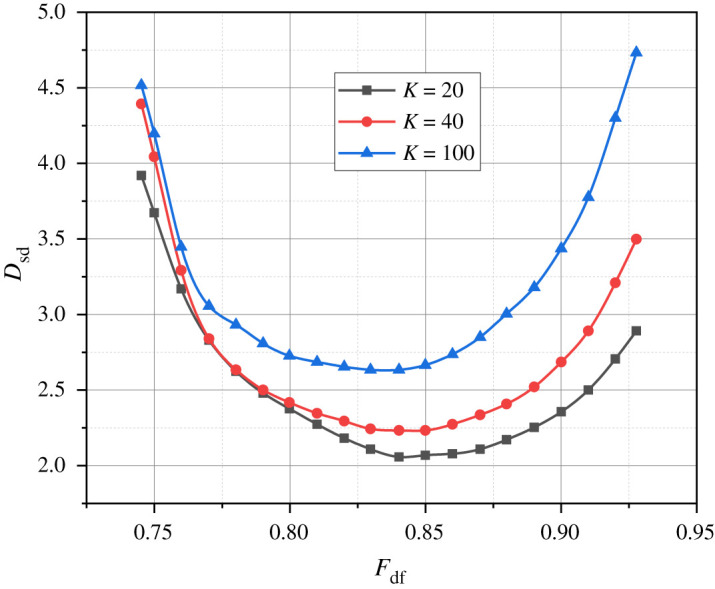
Results of the calculation of variants of the dependence *D*_sd_ = *f*(*F*_DF_) at *K* = {20, 40, 100}.

**Figure 4.**
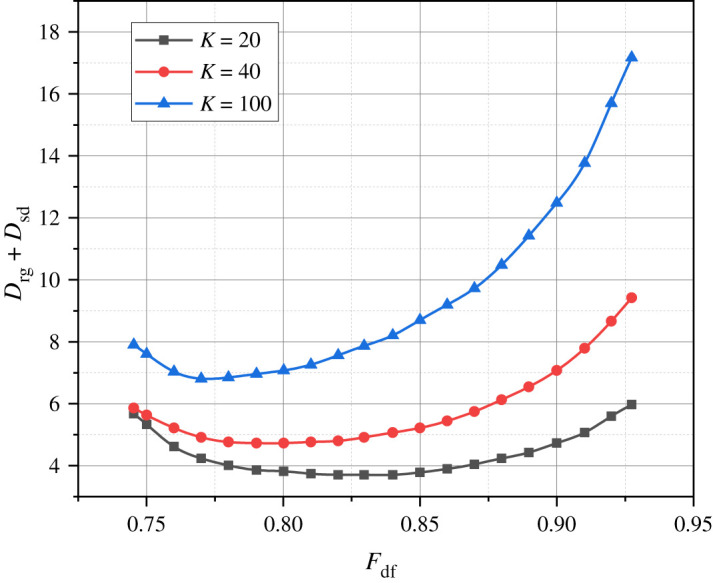
Results of the calculation of variants of the dependence *D*_rg_ + *D*_sd_ = *f*(*F*_DF_) at *K* = {20, 40, 100}.

Let's use the generalized expressions (2.22) and (2.3) along with the proprietary mathematical apparatus to calculate the corresponding analytical dependencies such as *D*_rg_ = *f*(*F_DF_*) (figure 2), *D*_sd_ = *f*(*F_DF_*) (figure 3), and *D*_rg_ + *D*_sd_ = *f*(*F*_DF_) (figure 4). The parameter *F*_DF_, which serves as the argument for all the just-introduced functional dependencies, is defined as *F*_DF_ = 8*S*/440.

Let's focus on the dependencies presented in figure 4, as the composite parameter *D*_rg_ + *D*_sd_ comprehensively characterizes the average duration of servicing an information message in the investigated WiMax-1/2 cluster.

All the graphs shown in figure 4 correspond to convex curves with pronounced extrema $F_{DF}^{*}$ in the visualized range of abscissa values. It is noteworthy that the increase in the argument of the dependency *D*_rg_ + *D*_sd_ = *f*(*F*_DF_) signifies a synchronous increase in the duration of the contention access frame. For all the graphs presented in Figure 6, the growth of abscissa values located to the right of the extrema is accompanied by a rapid increase in the average duration of time spent on the direct transfer of information messages in the investigated network. The explanation for this fact lies in the scenario where, at $F_{\mathrm{DF}}>F_{\mathrm{DF}}^{*}$, the volume of communication resources allocated by the base station for servicing an information message essentially becomes equal to the volume of communication resources allocated for serving the incoming stream of new information messages. Simultaneously, the reduction in the duration of the contention access frame leads to an increased likelihood of collisions (when multiple terminals attempt to use the same slot to transmit ACR requests). This, in turn, results in an increase in the registration time of information messages and the length of queues for the corresponding terminals. Additionally, it is worth noting that with an increase in the number of terminals in the investigated WiMax-1/2 cluster (*K* = {20, 40, 100}), the share of the contention access frame, denoted as *N* in the duration of the subinterval *D*_DF_ should be increased. For instance, when considering *K* = 20, this share should be 18%, and for *K* = 100, it should be 25%.

Therefore, the mathematical apparatus presented in §2 not only allows for the analysis of the process of direct transfer of information messages in the target WiMax-1/2 cluster in a compact and informative metric but also facilitates the optimization of this process while maintaining a fixed duration of the subinterval *D_DF_*.

## 4. Conclusion and future work

Rerouting of direct information traffic under the WiMax-1/2 technology control in the case of licensed frequency spectrum overload allows for ensuring communication continuity in the smart city's critical infrastructure. The support of such a process in the WiMax-1/2 cluster has its specificity, worthy of analytical formalization. In particular, it should be noted that reserving communication resources for the needs of target terminals in such a network is carried out using a random multiple-access algorithm.

The article presents a mathematical apparatus that allows estimating the average service duration of an information message during its transfer from the terminal to the WiMax-1/2 base station. Unlike analogues, the presented concept adequately describes the investigated process for any number of terminals, taking into account both the queuing effect on their side and the functioning of the cumulative query transmission mechanism inherent in WiMax-1/2 technology. Therefore, the proposed mathematical apparatus, describing the process of servicing an information message, takes into account both the average duration accompanied by potential collisions in the process of sending a request for the allocation of communication resources for its transmission to the base station and the average duration of the information message's stay in the terminal queue.

Experimental studies demonstrated the adequacy of the proposed mathematical apparatus for describing the investigated process. The experimental section also formulates the optimization problem of the investigated process resulting from the management of competitive access parameters.

Future research is planned to focus on expanding the range of optimization problem formulations for the process of direct information transfer in the WiMax-1/2 cluster and exploring computationally more efficient methods for solving systems of the form (1), taking into account the specificity of the research object.

## Data Availability

Most data is contained within the article. All the data are available on request due to restrictions, e.g. privacy or ethics. Supplementary material is available online [35].
